# Left ventricular geometric patterns in patients with type A aortic dissection

**DOI:** 10.1186/s12947-019-0152-4

**Published:** 2019-02-12

**Authors:** Soo-Jin Kim, Tae-Ho Park, Young-Rak Cho, Kyungil Park, Jong-Sung Park, Moo Hyun Kim, Young-Dae Kim

**Affiliations:** Department of Cardiology, Dong-A University Hospital, Daeshingongwon-Ro 26, Seo-gu, Busan, 49201 Republic of Korea

**Keywords:** Aorta, Dilatation, Left ventricular hypertrophy

## Abstract

**Background:**

Aortic dilatation is a major risk factor for aortic dissection. The aim of the present study was to assess the relationship between left ventricular (LV) geometry and maximal ascending aorta (MAA).

**Methods:**

We reviewed data from patients who were diagnosed with acute type A aortic dissection and who underwent surgical management from December 2002 to March 2016 at Dong-A University Hospital. Among 151 patients with non-Marfan aortic dissection in the study, 50 who had echocardiography preoperatively were investigated and MAA diameter was analyzed by LV geometric patterns.

**Results:**

Patients’ mean age was 59.6 ± 13.5 years and 38.0% were male. The mean MAA diameter was 52.9 ± 8.5 mm. MAA diameter was significantly correlated with LV mass index (r = 0.62, *P* < 0.001). On analysis by LV geometry, MAA diameter showed a significant difference between the 4 groups (*P* = 0.02), and the eccentric and concentric hypertrophy groups showed significantly larger MAA diameter than the other two groups.

**Conclusion:**

MAA diameter was associated with LV mass index and was significantly different between LV geometry types. In this study, not only concentric hypertrophy but also eccentric LV hypertrophy was related to larger MAA in type A aortic dissection patients.

## Introduction

Since type A aortic dissection is a life threatening condition requiring rapid diagnosis and treatment, preventive strategies are important [1–4]. Recent studies show that aortic size is related to the occurrence of aortic dissection events [5–8]. Aortic dilatation can be caused by decreased elastic fibers, vessel wall weakness, or shear force on the aortic vessel wall. It is also associated with high blood pressure, which causes morphological changes in the left ventricle (LV) such as concentric LV hypertrophy [7–12]. Although aortic dissection is known to be associated with LV hypertrophy, not all aortic dissection patients exhibit LV hypertrophy. Moreover, there is a lack of research on the relationship between different types of LV morphology and the risk of aortic dissection. Thus, in this study we evaluated the relationship between LV geometry and maximal ascending aorta (MAA) in patients with non-Marfan aortic dissection.

## Methods

We examined data from all patients who visited Dong-A University Hospital that had type A aortic dissection and underwent surgery from December 2002 to March 2016. Acute type A dissection was defined as any dissection involving the ascending aorta and/or aortic arch within 7 days after presenting with symptoms [13]. Data from a total of 161 patients were collected and patients’ clinical data, computed tomography (CT), and echocardiography were reviewed. One hundred fifty one patients were defined as non-Marfan syndrome patients who did not have connective tissue disease such as Marfan syndrome, Loeys-Dietz syndrome, or Ehlers-Danlos syndrome. We excluded patients with connective tissue diseases based on their medical records, but genetic examination for confirmation of the diagnosis was not performed. We measured the MAA diameter in 151 subjects who were examined by chest CT before the surgical intervention. LV geometry was evaluated in 50 patients who had echocardiography before the surgical intervention. We statistically analysed only the data of these 50 patients. Hypertension was defined as systolic blood pressure ≥ 140 mmHg or having had a history of hypertension, with or without ongoing pharmacologic treatment. All data were collected retrospectively and included information about patients’ demographic characteristics, medical history, clinical presentation, imaging findings, and clinical events, including 1-month mortality.

## Echocardiography

Transthoracic echocardiography was performed on patients before aortic surgery. Standard 2-dimensional and Doppler echocardiography were performed according to the recommendations of the American Society of Echocardiography [14]. LV ejection fraction (LVEF) was assessed using the modified Simpson method. LV end diastolic dimension (LVEDD) was measured at the chordae level. Interventricular septal thickness (IVST), posterior wall thickness (PWT) were measured at end-diastole. LV mass was estimated by LV cavity dimension and wall thickness at end-diastole, and LV mass index was adjusted to the body surface area. LV mass and relative wall thickness (RWT) were calculated using the following equations: LV mass (g) = 1.04 [(LVEDD + IVST + PWT)^3^ - LVEDD^3^)] × 0.8 + 0.6, RWT = 2 × PWT/LVEDD) [15]. LV mass index and RWT allowed further classification of LV geometry into 4 types. The cutoff for the LV mass index is 95 and 115 g/m^2^ for women and for men, respectively. Patients with an enlarged LV mass were classified as having concentric hypertrophy (RWT > 0.42) or eccentric hypertrophy (RWT > 0.42), while patients with normal LV mass were classified as having concentric remodeling (RWT > 0.42) or normal geometry (RWT > 0.42). Measurement of aortic parameters was performed at the levels of aortic valve annulus, sinuses of Valsalva, sinotubular junction, and proximal ascending aorta. Aortic valve annulus was measured at mid-systole phase using the zoom mode image. Sinus of Valsalva, sinotubular junction, and proximal ascending aorta were measured at end-diastole phase [14].

## Computed tomography

CT was performed using Aquilion 16, Aquilion ONE (Toshiba corporation, Tokyo, Japan). Non-enhanced and enhanced axial images and coronal images were obtained from the level of branching vessels of the aortic arch to the level below the iliac bifurcation, in slices with 2 mm to 5 mm thickness. MAA, the diameter of the maximally dilated portion of the ascending aorta, was defined as the largest value of the short-axial diameter of the ascending aorta and the perpendicular diameter of the curvature of aortic arch in this study, using retrospectively obtained CT images [16–19].

## Statistical analysis

Data analysis was performed using statistical analysis software (SPSS version 18). All data are presented as mean ± standard deviation for continuous variables, and as numbers with percentages for categorical variables. We used correlation and linear regression models for assessment of the association of LV mass index, aortic root size, age, and duration of hypertension, with MAA. We performed 1-way analysis of statistical variance with Bonferroni correction to compare the aortic root size, MAA dimension, and duration of hypertension between the 4 groups of LV geometry.

## Results

Baseline clinical characteristics of 50 non-Marfan patients with type A aortic dissection are shown in Table 1. The mean age was 59.6 ± 13.5 years, and 38% of the patients were male. Mean initial systolic blood pressure was 121.8 ± 25.2 mmHg and mean heart rate was 80.5 ± 16.8 beats per minute. The number of patients with underlying hypertension, diabetes mellitus, coronary artery disease, and atrial fibrillation were 38 (76.0%), 2 (4.0%), 8 (16.0%), and 8 (16.0%), respectively. The mean duration of hypertension was 6.6 ± 7.6 years. The symptoms of the patients were chest pain (*n* = 37, 74.0%), back pain (*n* = 12, 24.0%), dyspnea (*n* = 5, 10.0%), syncope or dizziness (*n* = 4, 8.0%), and headache (*n* = 1, 2.0%). Even though surgical management was performed, six patients (12.0%) died within 1 month after aortic dissection.

**Table 1:** Baseline characteristics (*n* = 50)

Gender (male)	19 (38.0%)
Age (years)	59.6 ± 13.5
Height (cm)	162.6 ± 8.0
Weight (kg)	63.9 ± 15.2
Body surface area (m^2^)	1.7 ± 0.2
SBP (mmHg)	121.8 ± 25.2
DBP (mmHg)	75.8 ± 15.9
Heart rate (beats/min)	80.5 ± 16.8
Diabetes mellitus	2 (4.0%)
Hypertension	38 (76.0%)
Chronic kidney disease	1 (2.0%)
Coronary artery disease	8 (16.0%)
Prior PCI or CABG	2 (4.0%)
Prior cerebrovascular accident	5 (10.0%)
Atrial fibrillation	3 (6.0%)
Dyslipidemia	2 (4.0%)
Smoking history	12 (24.0%)

Values are means ± SD or numbers with percentages. *SBP* systolic blood pressure, *DBP* diastolic blood pressure, *PCI* percutaneous coronary intervention, *CABG* coronary artery bypass graft

## Computed tomography and echocardiographic findings

**Fig. 1:**
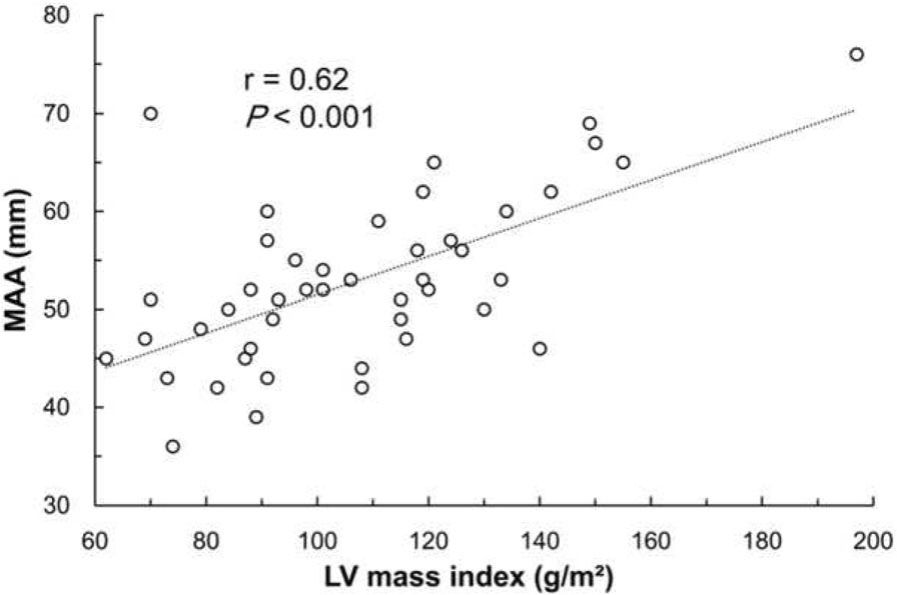
Correlations between LV mass index and MAA. MAA diameter was significantly correlated with LV mass index (r = 0.62, *P* < 0.001). LV, left ventricle; MAA, maximal ascending aorta. There are 5 overlapped dots in the figure

**Fig. 2:**
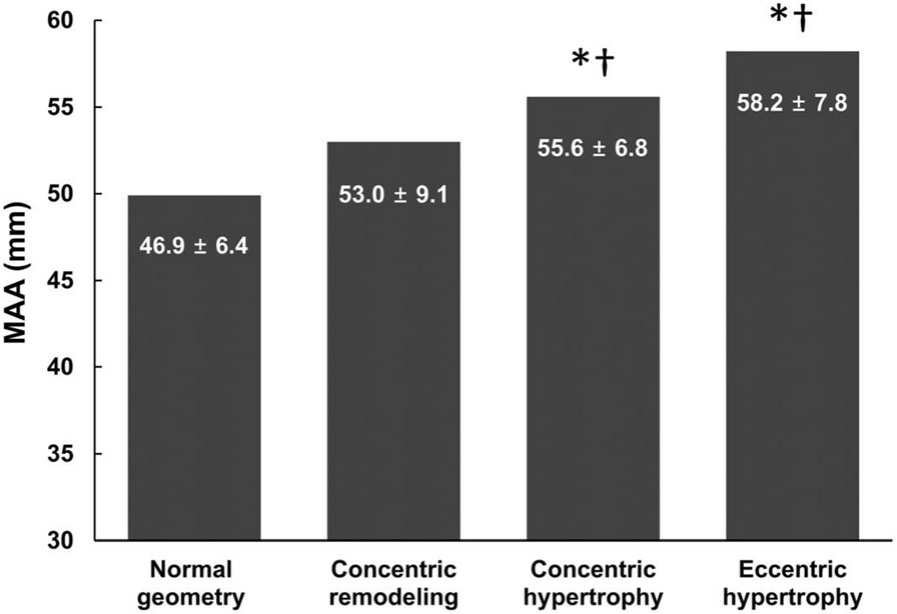
LV geometry and MAA. The MAA diameter was significantly different in the LV geometry groups; the eccentric and concentric hypertrophy groups showed significantly larger MAA diameter than the other two groups. LV, left ventricle; MAA, maximal ascending aorta. *P < 0.05 vs. normal geometry and †*P* < 0.05 vs. concentric remodeling

MAA diameter and echocardiographic parameters are presented in Table 2. The mean MAA diameter of the 50 patients who underwent preoperative echocardiography was 52.9 ± 8.5 mm. Linear regression analysis revealed that the MAA diameters were significantly correlated with the LV mass index (r = 0.62, *P* < 0.001, Fig. 1). More interestingly, the MAA diameter was significantly different in the 4 LV geometry groups (*P* = 0.02). The MAA diameter was 46.9 ± 6.4 mm, 53.0 ± 9.1 mm, 55.6 ± 6.8 mm, and 58.2 ± 7.8 mm in the normal, concentric remodeling, concentric LV hypertrophy, and eccentric LV hypertrophy groups, respectively. Among the groups of LV geometry, the eccentric and concentric groups showed significantly larger MAA diameter and LV mass index compared to the other groups (Fig. 2). Measurements of the aorta in different LV geometry groups are demonstrated in Table 3. The mean diameters of sinotubular junction (*P* = 0.022) and proximal ascending aorta (*P* = 0.011) in the eccentric LV hypertrophy group were significantly larger compared to those in the normal LV geometry group. MAA was significantly correlated with proximal ascending aortic dimension (r = 0.62, *P* < 0.001), sinotubular junction dimension (r = 0.55, *P* < 0.001) and sinus of Valsalva (r = 0.43, *P* = 0.003).

**Table 2:** MAA diameter and echocardiographic parameters

MAA diameter (mm)	52.9 ± 8.5
LVEF (%)	59.8 ± 9.8
LVEDD (mm)	49.3 ± 6.4
LV mass index (g/m^2^)	107.6 ± 26.8
Relative wall thickness (mm)	0.40 ± 0.10
Type of LV geometry	
Normal geometry	17 (34.0%)
Concentric remodeling	9 (19.6%)
Concentric hypertrophy	10 (20.0%)
Eccentric hypertrophy	14 (28.0%)
Aortic measurements	
Annulus (mm)	23.6 ± 2.7
Sinuses of Valsalva (mm)	39.7 ± 7.9
Sinotubular junction (mm)	37.0 ± 8.2
Proximal ascending aorta (mm)	47.3 ± 8.5

Values are means ± SD or numbers with percentages. *MAA* maximal ascending aorta, *LV* left ventricle, *LVEF* left ventricular ejection fraction, *LVEDD* left ventricular end diastolic diameter

**Table 3:** Aortic measurements according to LV geometry

	Normal geometry	Concentric remodeling	Concentric hypertrophy	Eccentric hypertrophy	*P*
Annulus (mm)	23.6 ± 2.6	21.8 ± 1.9	24.5 ± 3.0	24.0 ± 3.0	0.146
Sinuses of Valsalva (mm)	37.8 ± 4.6	36.9 ± 3.9	40.2 ± 5.2	43.4 ± 12.3	0.153
Sinotubular junction (mm)	34.2 ± 5.5	33.6 ± 3.3	37.4 ± 4.9	42.1 ± 12.0*	0.022
Proximal ascending aorta (mm)	43.2 ± 4.6	45.1 ± 8.1	49.1 ± 8.2	52.6 ± 10.0*	0.011

Values are means ± SD. *P < 0.05 vs. normal geometry. *LV* left ventricle

## Discussion

The major findings of the present study are as follows: (i) the MAA diameter was significantly different in the LV geometry groups (the eccentric and concentric hypertrophy groups showed significantly larger MAA diameter than the other two groups); and (ii) MAA was not severely dilated in non-Marfan patients with type A aortic dissection.

Previous studies have shown that aortic dilatation is related to a high risk of aortic dissection, although some studies insist that aortic diameter should be used as a predictor for aortic dissection only in patients with Marfan syndrome [5, 6, 17, 20, 21]. Hence, MAA diameter is widely used to evaluate the risk of aortic dissection. The current guideline recommends surgical management to Marfan syndrome patients with aortic aneurysm of the ascending aorta ≥50 mm and to non-Marfan syndrome patients with aortic aneurysm of the ascending aorta ≥55 mm [7]. However, a recent study reported that the MAA diameter at presentation of the aortic dissection was < 55 mm in non-Marfan syndrome patients with aortic dissection [6]. Likewise, patients in this study with non-Marfan aortic dissection had a mean MAA diameter of 52.9 mm, which is also smaller than the currently recommended criteria for surgery of 55 mm. More strikingly, only 25.2% of these patients with non-Marfan aortic dissection had a MAA ≥ 55 mm preoperatively.

Next to Marfan syndrome, hypertension is the most common predisposing factor of aortic dissection [1–3, 22–24]. In previous studies, high systolic blood pressure was shown to cause adaptive physiologic changes in the heart, known as concentric hypertrophy, through parallel replication of sarcomeres. In contrast, volume overload in the heart increases the end diastolic pressure and LV wall stress that leads to serial replication of sarcomeres, resulting in eccentric hypertrophy. Interestingly, after long-term exposure to high systolic pressure, pressure overload progresses to the point that the myocardium can no longer compensate through concentric hypertrophy, after which the myocardium undergoes fiber elongation, resulting in eccentric hypertrophy [10, 12]. LV mass index increases in cases of both concentric hypertrophy and eccentric hypertrophy. However, the LV mass index in patients with eccentric hypertrophy was slightly higher than that of the patients with concentric hypertrophy in this study. This may be one reason why patients with eccentric hypertrophy patients had slightly larger MAA diameters than did the patients with concentric hypertrophy.

In this study, among the LV geometry groups, the eccentric and concentric hypertrophy groups had a larger MAA diameter, which is the most potent predictor of aortic dissection, than the other groups.

The limitations of this study include that it was a single-center study and included only a small population of patients. Moreover, pre-operative echocardiography was not performed in many of the patients. The patients were not randomized, and the analysis of the data was done retrospectively.

## Conclusions

MAA diameter was associated with LV mass index and was significantly different between LV geometry types. Eccentric hypertrophy as well as concentric hypertrophy was related with larger MAA diameters in non-Marfan type A aortic dissection patients.

## Abbreviations

CT: Computed tomography
IVST: Interventricular septal thickness
LV: Left ventricle
LVEDD: Left ventricle end diastolic dimension
LVEF: Left ventricle ejection fraction
MAA: Maximal ascending aorta
PWT: Posterior wall thickness
RWT: Relative wall thickness
